# Sustained adherence to a Mediterranean diet and physical activity on all-cause mortality in the Melbourne Collaborative Cohort Study: application of the g-formula

**DOI:** 10.1186/s12889-019-7919-2

**Published:** 2019-12-26

**Authors:** Elizabeth J. Williamson, Julia Polak, Julie A. Simpson, Graham G. Giles, Dallas R. English, Allison Hodge, Lyle Gurrin, Andrew B. Forbes

**Affiliations:** 1 Department of Medical Statistics, London School of Hygiene & Tropical Medicine, London, WC1E 7HT UK; 2 Health Data Research UK (HDR UK), UK; 3 The Victorian Centre for Biostatistics (ViCBiostat), Melbourne, Victoria Australia; 4 Centre for Epidemiology and Biostatistics, Melbourne School of Population and Global Health, The University of Melbourne, Melbourne, Victoria Australia; 5 Cancer Epidemiology and Intelligence Division, Cancer Council Victoria, Melbourne, Victoria Australia; 6 Department of Epidemiology & Preventive Medicine, Monash University, Melbourne, Victoria Australia

**Keywords:** Time-varying confounding, G-methods, G-computation, Parametric G-formula

## Abstract

**Background:**

Adherence to a traditional Mediterranean diet has been associated with lower mortality and cardiovascular disease risk. The relative importance of diet compared to other lifestyle factors and effects of dietary patterns over time remains unknown.

**Methods:**

We used the parametric G-formula to account for time-dependent confounding, in order to assess the relative importance of diet compared to other lifestyle factors and effects of dietary patterns over time. We included healthy Melbourne Collaborative Cohort Study participants attending a visit during 1995–1999. Questionnaires assessed diet and physical activity at each of three study waves. Deaths were identified by linkage to national registries. We estimated mortality risk over approximately 14 years (1995–2011).

**Results:**

Of 22,213 participants, 2163 (9.7%) died during 13.6 years median follow-up. Sustained high physical activity and adherence to a Mediterranean-style diet resulted in an estimated reduction in all-cause mortality of 1.82 per 100 people (95% confidence interval (CI): 0.03, 3.6). The population attributable fraction was 13% (95% CI: 4, 23%) for sustained high physical activity, 7% (95% CI: − 3, 17%) for sustained adherence to a Mediterranean-style diet and 18% (95% CI: 0, 36%) for their combination.

**Conclusions:**

A small reduction in mortality may be achieved by sustained elevated physical activity levels in healthy middle-aged adults, but there may be comparatively little gain from increasing adherence to a Mediterranean-style diet.

## Background

The traditional Mediterranean diet is characterised by relatively high intakes of fruit, vegetables, cereals, grains, nuts, olive oil, and fish with a low intake of red meat and dairy products, and moderate consumption of alcohol, mainly as wine [1]. A randomised controlled trial of a Mediterranean-style diet with a primary outcome of cardiovascular disease (CVD) demonstrated reductions in cardiovascular events in the group randomised to the Mediterranean diet [2] and noted a reduction in all-cause mortality [3]. The NICE Guidelines [4] recommend following a Mediterranean-style diet post myocardial infarction (MI). A recent meta-analysis of cohort studies found that adherence to a Mediterranean diet was associated with a 10% reduction in CVD risk and an 8% lower risk of overall mortality for a 2-point increment in diet score [5]. More recent cohort studies have generally supported previous findings suggesting reductions in CVD mortality [6, 7] and all-cause mortality [6–9]. Similar associations were observed for men and women who had CVD at baseline [10]. Only one prospective study of healthy American adults, mostly men, found no evidence of an association between three different dietary indices, including a Mediterranean diet score, and mortality [11].

Adherence to a Mediterranean-style diet may reflect a tendency to healthier lifestyle choices in general, so it is important to assess the effect of diet in combination with other modifiable lifestyle factors, such as physical activity. Knoops et al. [12] found that increasing adherence to a healthful lifestyle, determined by combinations of smoking behaviour, alcohol consumption, physical activity, and adherence to a Mediterranean diet, was associated with a more than 50% reduction in all-cause mortality. Prinelli et al. found similar associations in a smaller study [9]. In a large American cohort, Mediterranean diet score was associated with a small (14%) reduction in mortality risk, with the combination of healthy diet, not smoking, healthy waist circumference and physical activity giving a relative risk of 0.27 (95% confidence interval: 0.25, 0.29) for mortality [8]. However, these studies considered only a single measurement of lifestyle factors at baseline, which may simply reflect the accumulation of a lifetime of healthy behaviours. To disentangle these separate effects it is necessary to consider changes in these lifestyle factors over time. However, with lifestyle exposures such as diet, confounders (e.g. diabetes) may change over time and may also be affected by prior exposure status; standard statistical methods cannot adequately address this type of confounding bias [13, 14]. Two studies analysed repeated dietary measurements [7, 10], but neither employed statistical methods accounting for potential time-dependent confounding by time-varying covariates.

We are unaware of any studies considering the combined effect of sustained diet and physical activity over time, accounting for time-dependent confounding. We use data from three waves of an Australian cohort study to investigate the individual and combined benefits of adherence to a Mediterranean-style diet and high physical activity levels on all-cause mortality over a 14-year period for middle-aged adults free of major disease, employing the parametric G-formula to account for time-dependent confounding.

## Methods

### Study population

The Melbourne Collaborative Cohort Study (MCCS) is a prospective study in Australia comprising 41,513 participants almost all of whom (99%) were aged between 40 and 69 years [15]. Participants were recruited from the community between 1990 and 1994. Participants attended a study centre at recruitment (T0), where physical measurements, interviewer-administered questionnaires and blood samples were completed. Participants were followed up approximately 5 years later by mail (T1; 1995–1999, approx. 87% response proportion) and subsequently attended a study centre approximately 7 years later (T2; 2003–2007, approx. 66% response proportion) where physical measurements, blood samples and interviewer-administered questionnaires were repeated. The Cancer Council Victoria’s Human Research Ethics Committee approved the study.

MCCS participants were eligible for the present study if they completed the questionnaire at the first follow-up (T1), and were free of major disease (cancer, stroke, heart attack, bypass, angioplasty, diabetes) at that time. Participants with extreme self-reported dietary intakes at recruitment (top and bottom 1%) were excluded. T1 is taken as the baseline for the current study, rather than recruitment (T0), to allow careful adjustment for previous dietary and physical activity patterns. Migrants from Southern Europe were excluded due to high attrition, resulting in too small a sample to draw reliable conclusions for this subgroup.

### Dietary data

A 121-item food frequency questionnaire was developed for the study [16] and was self-administered at recruitment (T0). Diet was assessed by a shorter self-administered questionnaire at the interim follow-up (T1) and an extended questionnaire at the final visit (T2). Good validity and reliability of the two longer questionnaires has been demonstrated [17–19]. Alcohol consumption was measured using beverage-specific frequency and quantity questions. A Mediterranean Diet Score (MDS) was calculated following Trichopoulou et al. [1] with the alcohol component modified to adapt to the Australian context. For each wave, participants were allocated: 1 point for intakes of vegetables, fruit, cereals, legumes and fish above (or equal to) sex-specific medians; 0 points for intakes below the median. For dairy and red meat, this was reversed (1 point for intakes below the median; 0 otherwise). For alcohol, 1 point was allocated for 5–20 g/d for women, and 10–20 g/d for men (a lower limit than the original scoring, following Australian national guidelines [20]); lower or higher intakes scored zero. We replaced the ratio of monosaturated:saturated fats [1] by olive oil intake, since red meat is an important source of monosaturated fat in Australia [21]. The final score, calculated at each of the three study time-points (T0, T1, T2), ranged from 0, indicating no adherence to a Mediterranean style diet, to 9 indicating high adherence to a Mediterranean diet. Scores were categorised into three groups: low (0–3), moderate (4, 5) and high (6–9) adherence to a Mediterranean diet; these categorised scores are used throughout.

### Physical activity data

Physical activity was assessed by questions regarding the amount and intensity of regular exercise undertaken. Responses were aggregated to an overall score by weighting responses based on relative energy expenditure of different activities. The questions asked at the three waves differed, thus the absolute activity scores are not comparable across time-points. The questions in the first two waves were obtained from the Risk Factor Prevalence Study conducted by the National Heart Foundation and Australian Institute of Health, and have been shown to have predictive validity for current and subsequent obesity [22]. The third wave used the validated long form International Physical Activity Questionnaire [23]. The scores were categorised into low, moderate and high physical activity groups; cut-offs were chosen to represent comparable activities at the three waves: the low-activity category represents at most walking or undertaking moderate exercise twice a week; the high-activity category represents doing intensive activity (e.g. running) three or more times a week, or intensive activity twice a week combined with daily walking or moderate activity. We consider the categorised activity scores throughout.

### Mortality, cancer and cardiovascular disease

Deaths were identified through the Victorian Registry of Births, Deaths and Marriages, and the National Death Index. Death records were complete to April 2011. High sensitivity and specificity of linkage to the National Death Index has been demonstrated [24]. Cancer diagnoses were identified from notifications to the Victorian Cancer Registry. Participants who attended a study centre at the third wave (T2) provided information about heart attack, angioplasty, bypass or stroke since the previous wave. Questions about diabetes and angina diagnoses were added during data collection in the third wave (T2) thus this information is not available for all participants. For participants who did not attend the third wave, the intended date of follow-up – conservatively estimated as the last date of T2 follow-up – was used to determine whether deaths occurred pre or post T2.

### Potential confounders

A causal diagram was drawn to inform the selection of potential confounders (Additional file 1: Figure S1). Weight was measured according to standard procedures [25] at recruitment (T0) and (T2), with self-reported weight at the postal follow-up (T1). These weights were used to calculate body mass index (BMI, kg/m^2^) at each wave, using the height measured at recruitment, grouped as: underweight < 18.5, normal 18.5- < 25, overweight 25- < 30, obese I 30- < 35, obese II/III 35+. Other confounders considered were: sex, education (primary school, some high/technical school, completed high/technical school, degree/diploma), smoking status (never, former, current), history of angina, arthritis or asthma, family history of heart attack (parent or sibling), living alone, total plasma cholesterol (mmol/L), and hypertension (SBP > 140 mmHg or DBP > 90). Where age (at T1) was categorised, it was grouped as: < 50, 50–55, 55–60, 60–65, 65–70, 70+ yrs. Alcohol consumption forms part of the Mediterranean diet score, thus was not considered a confounder at T1. Previous consumption (at T0) was a potential confounder, grouped as: abstainer, former drinker, low (< 40 g/d men, < 20 g/d women), medium (40–60 g/d men, 20–40 g/d women), and high (> 60 g/day men, > 40 g/d women). At the second clinic visit (T2), the values of living alone, smoking status, BMI, and plasma cholesterol were updated.

### Statistical analysis

Unadjusted Kaplan-Meier failure curves for mortality by Mediterranean diet and physical activity scores (at T1) were drawn. For comparison with previous studies, Cox regression, with an age timescale, was used to estimate hazard ratios for mortality associated with Mediterranean diet and physical activity scores, with follow-up from the date of the T1 questionnaire to the first of death or 1st April 2011, adjusting for all confounders above. Initial models considered exposure and confounders fixed at T1; subsequent models time-updated exposures, confounders and comorbidities at T2. Multiple imputation was used to handle missing data (details in Additional file 1: Box S1). The proportional hazards assumption was checked using Schoenfeld residual tests.

To account for time-varying confounders (BMI, smoking, living alone, cholesterol, and comorbidities developed in the intervening time) we applied the parametric G-formula (see [26–28] for similar applications). Briefly, the parametric G-formula is a natural extension of conventional direct standardisation of mortality risks, accounting for time-dependent confounding. It estimates risks under population-wide exposure to a sequence of exposure values, often referred to as ‘hypothetical interventions’. The standardised cumulative risk is estimated by a weighted average of the all-cause mortality risk under the given intervention and observed baseline patient characteristics. The weights reflect the distribution of time-varying confounders and are estimated via parametric regression models. The weighted average is approximated by Monte Carlo simulation. Details are given in Additional file 1: Box S2.

We considered a number of ‘hypothetical interventions’ on diet and physical activity levels. Some involved intervening only at T1 (our study baseline), thereafter allowing diet and physical activity to evolve naturally; others involved repeating the intervention at T2 (approx. 7 years later). We considered interventions that set either the physical activity score or the Mediterranean Diet score, or both, to the highest level. These were compared with two control interventions: (i) setting physical activity and Mediterranean diet scores to the lowest level at T1 and T2, and (ii) setting all to the middle level. All interventions were also repeated restricted to the subpopulation of participants whose BMI at the time of the intervention fell into the obese range (≥ 30 kg/m^2^). Finally, we estimated the mortality risk under the natural evolution of physical activity and diet scores (the ‘natural course’). We estimated all cumulative mortality risks under no loss to follow-up.

Non-parametric bootstrapping was performed within each imputed dataset, applying the parametric G-formula then using Rubin’s rules to obtain normal-based 95% confidence intervals and overall estimates of cumulative mortality risks. The risk ratio and risk difference, compared with the natural course (i.e. no intervention, no loss to follow-up), are reported.

Sensitivity analyses included changing the order of time-varying confounders, intermediate comorbidities and the exposures of interest. Split-sample cross validation with 500 replications was used to assess out-of-sample predictive ability for models for mortality, diet, and physical activity scores, comparing models using splines, categories and polynomial modelling of continuous predictors.

All analyses were performed using Stata version 14.1 (Stata Corp, Texas, United States).

## Results

### Sample description

Of 22,213 participants of the Melbourne Collaborative Cohort Study eligible for our study, 18,386 (82.8%) had complete information at T1 (our study baseline, Additional file 1: Figure S2). The median age at study baseline was 58, and 13,980 (62.9%) participants were female (Table 1).

**Table 1:** Description of study sample (*n* = 22,213)

Characteristics of participants at baseline ^a^		Women (*n* = 13,980)		Men (*n* = 8233)	
	N_miss_^b^	*N*	%	*N*	%
Age (years; Median, IQR)		58.3	(50.7, 66.3)	57.3	(50, 65.9)
Education:					
Primary school		4,65	3.3%	224	2.7%
Some high school		6906	49.4%	2636	32%
Completed high school		2962	21.2%	2278	27.7%
Completed tertiary		3647	26.1%	3095	37.6%
Alcohol consumption^c^:					
Lifetime abstainer		4228	30.2%	982	11.9%
Former drinker		438	3.1%	354	4.3%
Low (women: < 20 g/d, men: > 40 g/d)		7391	52.9%	5654	68.7%
Medium (women: 20-40 g/d, men: 40-60 g/d)		1489	10.7%	718	8.7%
High (women: 40 + g/d, men: 60 + g/d)		434	3.1%	525	6.4%
SBP > 140 or DBP > 90	37	4314	30.9%	3224	39.2%
Self-reported history of:					
Angina	21	336	2.4%	188	2.3%
Asthma	67	2382	17.1%	1193	14.5%
Arthritis^c^		4675	33.4%	1677	20.4%
Family history of heart attack		6004	42.9%	3154	38.3%
Cholesterol (mmol/L; median (IQR))^c^	57	5.4	(4.8, 6.2)	5.4	(4.7, 6.0)
Living alone	249	2968	21.5%	1092	13.4%
BMI (kg/m^2^; median (IQR))	1513	24.7	(22.4, 27.7)	25.9	(24, 28.1)
Smoking:	19				
Never		9155	65.6%	4063	49.4%
Former		3903	27.9%	3556	43.2%
Current		908	6.5%	609	7.4%
Mediterranean Diet score^d^:	2504				
Low (0–3)		4095	33.1%	3157	42.9%
Medium (4–5)		5158	41.7%	2936	39.9%
High (6–9)		3105	25.1%	1258	17.1%
Physical activity^e^:	388				
Low		5073	37.0%	2661	32.8%
Medium		6310	46.0%	3348	41.2%
High		2324	17.0%	2109	26.0%

^a^ Study baseline is T1 (1995–1999); ^b^ Number of missing values; ^c^ Measured at T0 (1990–1994). IQR – inter-quartile range (i.e. 25th – 75th percentile). ^d^MDS groups: low (scores 0–3), medium (4–5) and high (6–9); ^e^Physical activity groups: low (e.g. walking twice a week or less), medium (e.g. jogging twice a week), high (e.g. running three or more times a week)

There was considerable within-participant change in diet and activity scores over the three measurement occasions (data not shown), with all possible patterns over time represented in the data. There were no substantial changes in the mean BMI or cholesterol over time (Additional file 1: Table S1), and the proportion of participants living alone increased over time, particularly for women, while the number of current smokers decreased.

During a median follow-up of 13.6 years (25th – 75th percentile: 13.2, 14.1), 2163 (9.7%) participants died. Of these, 1018 (4.6%) died before the T2 follow-up visit and 1145 (5.4%) died between the T2 visit and the end of follow-up (Additional file 1: Table S2). Notably, the observed mortality risk post-T2 for the whole sample (5.4%) was higher than the mortality risk for those with T2 follow-up data (4.2%), suggesting that those lost to follow-up were less healthy.

### Cox regression models

**Fig. 1:**
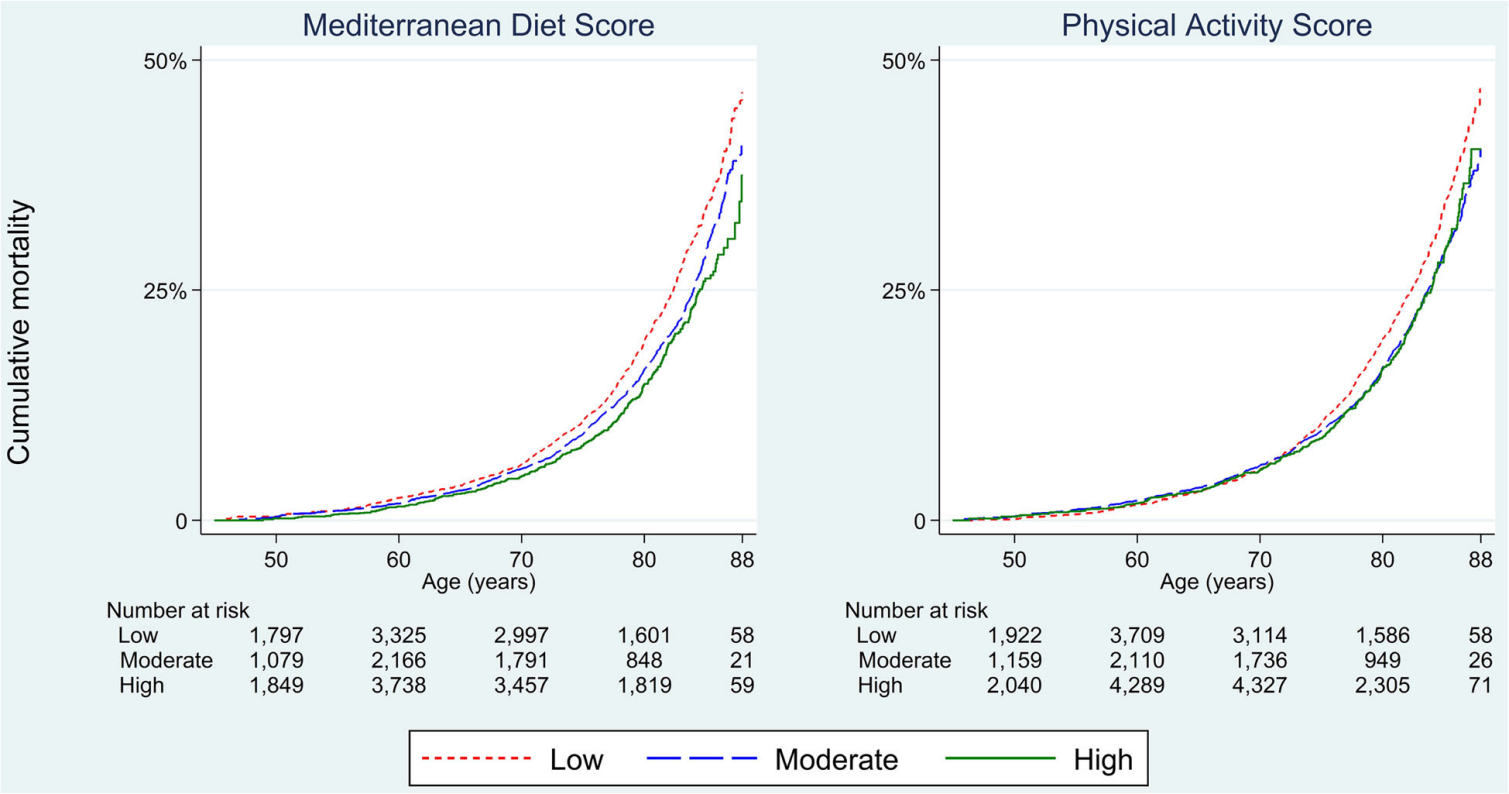
Kaplan-Meier failure estimates for all-cause mortality

Unadjusted Kaplan-Meier plots (Fig. 1) showed a pattern of lower Mediterranean diet and physical activity scores corresponding to higher mortality.

Cox regression models showed lower hazards of mortality associated with higher Mediterranean diet and physical activity scores (Table 2), with stronger associations seen for physical activity. Accounting for previous diet and physical activity attenuated the estimated hazard ratios; time-updating all measurements resulted in stronger hazard ratios. A model with time-updated exposures accounting for previous diet and physical activity gave a hazard ratio of 0.81 (95% confidence interval (CI): 0.70, 0.93) for high versus low adherence to a Mediterranean-style diet, and a hazard ratio of 0.71 (95% CI: 0.62, 0.81) for high versus low physical activity scores. Complete case analysis gave similar estimates (Additional file 1: Table S4).

**Table 2:** Hazard ratios (HR) for all cause mortality from Cox models, for Mediterranean diet score (MDS) and physical activity (PA)^a^

	Mediterranean Diet Score^b^				Physical Activity Score^c^			
	HR	95% CI		*P*	HR	95% CI		*P*
Single time-point exposure (at T1)								
Low	Ref				Ref			
Medium	0.90	(0.82,	1.00)	0.051	0.84	(0.76,	0.92)	< 0.001
High	0.89	(0.77,	1.01)	0.078	0.78	(0.68,	0.88)	< 0.001
+ adjusting for previous MDS & PA (T0)								
Low	Ref				Ref			
Medium	0.92	(0.83,	1.03)	0.137	0.85	(0.77,	0.94)	0.002
High	0.92	(0.80,	1.07)	0.285	0.81	(0.70,	0.93)	0.003
Time-updated MDS & PA								
Low	Ref				Ref			
Medium	0.87	(0.78,	0.96)	0.005	0.74	(0.66,	0.83)	< 0.001
High	0.79	(0.69,	0.90)	0.001	0.69	(0.61,	0.79)	< 0.001
+ adjusting for previous MDS & PA								
Low	Ref				Ref			
Medium	0.88	(0.79,	0.97)	0.012	0.75	(0.67,	0.84)	< 0.001
High	0.81	(0.70,	0.93)	0.003	0.71	(0.62,	0.81)	< 0.001

^a^Missing data were handled using multiple imputation. Study baseline is T1 (1995–1999), Initial cohort (MCCS) recruitment is T0 (1990–1994). ^b^MDS groups: low (scores 0–3), medium (4–5) and high (6–9); ^c^Physical activity groups: low (e.g. walking twice a week or less), medium (e.g. jogging twice a week), high (e.g. running three or more times a week)

### Parametric G-formula

The parametric G-formula estimated the overall cumulative mortality to be 9.88% (95% CI: 9.47, 10.28%) under the natural course, closely replicating the observed mortality risk of 9.74% (Table 3).

**Table 3:** Estimated cumulative mortality risks per 100 people associated with hypothetical interventions on physical activity (PA) and Mediterranean diet score (MDS)

	Pre-T2 mortality risk (95% CI)^c^		Post-T2 mortality risk (95% CI)^c^		Total^a^ mortality risk (95% CI)^c^		Risk difference^b^ (95% CI)^c^		Risk Ratio^b^ (95% CI)^c^		PAF (95% CI)^c^	
Observed risks												
Among whole cohort	4.58	(4.31, 4.86)	5.40	(5.10, 5.71)	9.74	(9.35, 10.13)						
Among followed-up^d^	4.58	(4.31, 4.86)	4.16	(3.79, 4.53)	8.55	(8.12, 8.99)						
Estimated risks under no intervention												
Natural course	4.59	(4.30, 4.87)	5.54	(5.22, 5.87)	9.88	(9.47, 10.28)	Ref		Ref			
Estimated risks under hypothetical interventions^e^												
Intervene on everyone												
*Control interventions (T1 and T2)*												
Low PA & low MDS	5.68	(4.87, 6.50)	7.66	(6.38, 8.94)	12.90	(11.54, 14.27)	3.03	(1.75, 4.30)	1.31	(1.18, 1.44)	−0.31	(−0.44, − 0.18)
Medium PA & MDS	4.53	(3.87, 5.19)	4.66	(3.79, 5.52)	8.97	(7.97, 9.98)	−0.90	(−1.86, 0.06)	0.91	(0.81, 1.01)	0.09	(−0.01, 0.19)
Low PA	5.15	(4.63, 5.68)	7.10	(6.18, 8.03)	11.89	(10.90, 12.88)	2.01	(1.13, 2.89)	1.20	(1.11, 1.29)	−0.20	(− 0.29, − 0.11)
Low MDS	4.63	(4.08, 5.17)	6.39	(5.68, 7.10)	10.72	(9.88, 11.55)	0.84	(0.15, 1.53)	1.09	(1.02, 1.15)	− 0.09	(− 0.15, − 0.02)
*Single interventions (T1)*												
High PA	4.35	(3.69, 5.00)	4.94	(4.19, 5.70)	9.08	(8.12, 10.03)	−0.80	(−1.67, 0.07)	0.92	(0.83, 1.01)	0.08	(−0.01, 0.17)
High MDS	4.71	(3.94, 5.49)	5.18	(4.44, 5.93)	9.65	(8.67, 10.63)	−0.22	(−1.13, 0.68)	0.98	(0.89, 1.07)	0.02	(−0.07, 0.11)
High PA & MDS	4.63	(3.24, 6.03)	4.40	(3.04, 5.77)	8.83	(6.95, 10.71)	−1.05	(−2.90, 0.81)	0.89	(0.71, 1.08)	0.11	(−0.08, 0.29)
*Repeated interventions (T1 and T2)*												
High PA	4.35	(3.69, 5.00)	4.39	(3.58, 5.19)	8.54	(7.56, 9.53)	−1.33	(−2.23, − 0.43)	0.87	(0.77, 0.96)	0.13	(0.04, 0.23)
High MDS	4.71	(3.94, 5.48)	4.73	(3.93, 5.54)	9.22	(8.17, 10.27)	−0.65	(−1.64, 0.33)	0.93	(0.83, 1.03)	0.07	(−0.03, 0.17)
High PA & MDS	4.63	(3.24, 6.03)	3.59	(2.22, 4.96)	8.06	(6.25, 9.87)	−1.82	(−3.60, −0.03)	0.82	(0.64, 1.00)	0.18	(0.00, 0.36)
Intervene on obese only												
*Control interventions (T1 and T2)*												
Low PA & MDS	4.73	(4.42, 5.03)	5.86	(5.48, 6.24)	10.31	(9.85, 10.77)	0.43	(0.24, 0.62)	1.04	(1.02, 1.06)		
Medium PA & MDS	4.56	(4.26, 4.87)	5.33	(4.99, 5.67)	9.65	(9.23, 10.08)	−0.22	(−0.40, −0.05)	0.98	(0.96, 1.00)		
Low PA	4.66	(4.36, 4.95)	5.75	(5.40, 6.09)	10.14	(9.71, 10.56)	0.26	(0.13, 0.39)	1.03	(1.01, 1.04)		
Low MDS	4.60	(4.31, 4.90)	5.67	(5.33, 6.02)	10.02	(9.59, 10.44)	0.14	(0.02, 0.26)	1.01	(1.00, 1.03)		
*Single interventions (T1)*												
High PA	4.54	(4.25, 4.83)	5.45	(5.11, 5.78)	9.74	(9.31, 10.17)	−0.14	(−0.30, 0.02)	0.99	(0.97, 1.00)		
High MDS	4.60	(4.29, 4.90)	5.51	(5.16, 5.86)	9.85	(9.42, 10.29)	−0.02	(− 0.19, 0.14)	1.00	(0.98, 1.01)		
High PA & MDS	4.58	(4.23, 4.92)	5.36	(5.00, 5.73)	9.70	(9.22, 10.17)	−0.18	(−0.46, 0.10)	0.98	(0.95, 1.01)		
*Repeated interventions (T1 and T2)*												
High PA	4.54	(4.25, 4.83)	5.29	(4.95, 5.64)	9.59	(9.17, 10.02)	−0.28	(−0.45, − 0.12)	0.97	(0.95, 0.99)		
High MDS	4.60	(4.28, 4.91)	5.39	(5.04, 5.75)	9.74	(9.30, 10.18)	−0.14	(−0.33, 0.06)	0.99	(0.97, 1.01)		
High PA & MDS	4.58	(4.23, 4.93)	5.15	(4.78, 5.52)	9.49	(9.01, 9.97)	−0.38	(−0.67, − 0.10)	0.96	(0.93, 0.99)		

*PA* Physical activity, *MDS* Mediterranean Diet Score, *PAF* population attributable fraction, *CI* confidence interval. T1 is study baseline is (1995–1999), T0 is initial cohort (MCCS) recruitment (1990–1994), T2 is follow-up (2003–2007). ^a^ Estimated as: Pr (Dead) = Pr (Dead pre-T2) + Pr (Dead post-T2|Alive at T2) x Pr (Alive at T2); ^b^ Compared with natural course; ^c^ 95% normal-based bootstrap confidence intervals using 100 bootstrap samples per imputed dataset; ^d^ Death post-T2 estimated using participants followed-up at T2 – the estimator we would obtain if outcome data were available only on followed-up participants, as is often the case. ^**e**^MDS groups: low (scores 0–3), medium (4–5) and high (6–9); Physical activity groups: low (e.g. walking twice a week or less), medium (e.g. jogging twice a week), high (e.g. running three or more times a week)

Hypothetical interventions repeatedly setting physical activity to the highest level (T1 and T2) resulted in a larger decrease (RR 0.87, 95% CI: 0.77, 0.96), compared to the equivalent intervention on the Mediterranean diet score, RR 0.93 (95% CI: 0.83, 1.03), with RR 0.82 (95% CI: 0.64, 1.00) for their combination. On the risk difference scale, this latter combined repeated intervention was estimated to result in an absolute reduction in all-cause mortality of 1.82 deaths per 100 people (95% CI: 0.03, 3.6). Interventions only on participants classed as obese showed similar trends, although the overall risk differences and risk ratios were much closer to the null.

The population attributable fraction was estimated as 13% (95% CI: 4, 23%) for sustained high physical activity, 7% (95% CI: − 3, 17%) for sustained high Mediterranean diet score and 18% (95% CI: 0, 36%) for their combination.

Complete case analysis and changing the order of comorbidities, the time-varying confounders, physical activity and Mediterranean diet scores made no material difference to the estimates (see Additional file 1: Tables S5-S8).

## Discussion

Both sustained high physical activity and high adherence to a Mediterranean-style diet were associated with reduced all-cause mortality. We estimated that the population attributable fractions associated with sustained high physical activity and high adherence to a Mediterranean-style diet were: 13% (95% CI: 4, 23%) and 7% (95% CI: − 3, 17%), respectively.

### Comparison with other studies

In contrast to our analysis, which did not find strong evidence of a benefit for the Mediterranean diet score, previous analyses of the MCCS found evidence of mortality benefits in many groups (e.g. [29–32]); these analyses, however, looked only at diet at study recruitment (T0). This may suggest that diet works over the long term to reduce risk and that what one does aged 50–65 or 60–75 years is not as important as what one did at 45–60 years (ages are approximate inter-quartile ranges at the three waves). However, the PREDIMED and Lyon Diet Heart Study provide evidence of short-term (4–5 years) cardiovascular benefit of changing to a more Mediterranean-style diet [2, 33]. It may be that benefits to all-cause mortality take longer to occur. Alternatively, although we included only “healthy” participants at our study baseline (T1), we cannot exclude reverse causation by unmeasured adverse health events, which contributed to subsequent mortality risk, potentially attenuating the association with change in Mediterranean diet score.

Behrens et al. [8] evaluated four risk factors (abdominal leanness, non-smoking, recommended physical activity and adherence to a Mediterranean diet), alone and combined, in relation to all-cause mortality in the NIH-AARP Diet and Health Study including 170,000 American men and women. Their population attributable fractions were 10% (95% CI: 8, 11%) for adhering to a Mediterranean-style diet and 5% (95% CI: 4, 6%) for meeting recommendations for physical activity. While these are of similar magnitude those found in our study, the benefit from a Mediterranean-style diet was relatively greater than for physical activity, in contrast to our observations. Tong et al. [7] report a population attributable fraction for their Mediterranean-style diet of 5.4% (95% CI: 1.3, 9.5%) for all-cause mortality but did not study the contribution of physical activity. These attributable fractions cannot be compared directly across studies because figures depend on the prevalence of the risk factors in each study and these varied across these studies.

Prinelli et al. [9] looked at the contributions of a Mediterranean diet, not smoking and being physically active to risk of all-cause mortality in a small Italian cohort. Each of the factors was associated with reduced risk, and the benefit was cumulative as more of the healthy lifestyle behaviours were adhered to, with adherence to all three being associated with a 73% reduced risk of death. Behrens et al. [8] similarly report a cumulative benefit for low-risk behaviours in diet, physical activity and smoking that together were estimated to be associated with 33% of deaths; while Knoops et al. [12] found adherence to Mediterranean diet, moderate alcohol consumption, physical activity and non-smoking could avert 60% of all deaths. We found that adherence to MDS and physical activity recommendations together was better than either alone but the population attributable fraction was only 19%. These observations suggest that making improvements across multiple behaviours is likely to provide the greatest benefit, and highlight the contribution of not smoking to risk reduction.

### Strengths and limitations

Unlike most other observational studies looking at the association between Mediterranean diet score and mortality, we were able to include repeated measures of diet and physical activity over time, but we had only two post-baseline measurement occasions. Ideally, multiple repeated measurements of confounders and exposures would be available with shorter gaps in between, to fully address the question of sustained adherence to particular dietary and activity habits.

While we adjusted carefully for confounders, we cannot rule out the possibility of residual confounding bias. For example, family and work environments are factors which may affect mortality but we were not able to measure these. However, we did not think these likely to be strong confounders a priori. If a Mediterranean style diet were more expensive than a more traditional Australian diet, then the individuals who chose to follow a more Mediterranean-style diet might have been of a higher socioeconomic status. We attempted to mitigate this by adjusting for education. In addition, current evidence suggest little evidence of such a price difference [34].

Our analysis makes an assumption called consistency, which requires all ways of achieving a particular Mediterranean diet score or activity score to be comparable in their effects on mortality. However, different types of diets can achieve the same Mediterranean diet score, which makes the interpretation of effects of high adherence to this style of diet ambiguous. This, however, is a limitation in any attempt to attach a causal interpretation to associations between Mediterranean diet scores and health outcomes.

This parametric G-formula has been criticised due to the potential for bias through model misspecification, due to the reliance on the specification of a large number of regression models. We attempted to mitigate this possibility by a range of model validation techniques and a number of sensitivity analyses. We were able to replicate closely the observed mortality risks using our complex system of predictive equations, providing some assurance of adequate model specification. The G-formula is also subject to the G-null paradox: essentially, the null hypothesis (no effect of diet/physical activity on mortality) will always be rejected in sufficiently large samples [13]. However, these biases caused by non-compatibility of the models within the G-formula are likely to be small compared with other sources of potential bias, such as measurement error or residual confounding.

Information about physical activity and diet were obtained via self-report. It is plausible that measurement error is non-differential with respect to our outcome, all-cause mortality, especially since the analysis was restricted to a fairly healthy population at our study baseline.

We modified the original Mediterranean Diet Score [1], using a lower upper limit for alcohol in order to reflect Australian national guidelines, and replacing the ratio of monosaturated:saturated fats by olive oil intake. Using olive oil as the main fat source is a key feature of the Mediterranean Diet [35], and we believe this best reflects the beneficial components of a Mediterranean diet. In a previous study using MCCS data we reported that across three categories of MDS calculated the same way, there was wide variation in olive oil intake and little variation in saturated fat intake [32], suggesting we have captured this aspect appropriately. Changing the alcohol limit is unlikely to have had much impact due to the small number of individuals in the study consuming large quantities of alcohol.

Strengths of our study include the large sample, repeated measurement of rich confounder and exposure data, linkage of the whole sample to outcome data through existing registries, and careful application of an analysis approach specifically designed to address questions concerning sustained adherence to exposures over time, avoiding problems due to measured time-dependent confounding.

## Conclusions

Our results suggest a small overall reduction in mortality may be achieved by sustained elevated physical activity levels across the population of healthy middle-aged adults, but there may be comparatively little gain from increasing adherence to a Mediterranean-style diet. These results contribute to the body of evidence regarding associations between Mediterranean diet, physical activity and mortality. Similar analyses using data from studies with a larger number of repeated measurements of diet and physical activity over time would further add to our understanding of the comparative benefits of these aspects of a healthful lifestyle.

## Supplementary information

**Additional file 1: Figure S1.** Causal diagram for study. **Figure S2.** Flowchart for study participants. **Table S1.** Summary of time-varying characteristics in study sample. **Table S2.** Comorbidities and mortality during follow-up. **Table S3.** Missing data at the three waves. **Table S4.** Compete case analysis – Cox models for all cause mortality. **Table S5.** Complete case analysis – All-cause mortality risks using the G-formula. **Table S6.** G-formula sensitivity analysis (re-ordering time-varying comorbidities). **Table S7.** G-formula sensitivity analysis (re-ordering time-varying confounders). **Table S8.** G-formula sensitivity analysis (re-ordering intervention variables). **Box S1.** Details of imputation approach for missing data. **Box S2.** Overview of the parametric G-formula.

## Data Availability

Data are available in collaboration via PEDIGREE (http://www.pedigree.org.au/) to scientifically meritorious and ethically approved Australian and international research proposals.

## Abbreviations

BMI: Body Mass Index
CI: Confidence Interval
CVD: Cardiovascular disease
DBP: Diastolic blood pressure
HR: Hazard Ratio
IQR: Interquartile range
MCCS: Melbourne Collaborative Cohort Study
MDS: Mediterranean Diet Score
MI: Myocardial Infarction
PA: Physical Activity
PAF: Population attributable fraction
SBP: Systolic blood pressure

## References

[1] Trichopoulou A, Costacou T, Bamia C (2003). Adherence to a Mediterranean diet and survival in a Greek population. N Engl J Med.

[2] de Lorgeril M, Salen P, Martin JL (1999). Mediterranean diet, traditional risk factors, and the rate of cardiovascular complications after myocardial infarction: final report of the Lyon diet heart study. Circulation.

[3] de Lorgeril M, Salen P, Martin JL (1998). Mediterranean dietary pattern in a randomized trial: prolonged survival and possible reduced cancer rate. Arch Intern Med.

[4] National Institute for Health and Care Excellence. Myocardial Infarction: cardiac rehabilitation and prevention of further cardiovascular disease: NICE; 2013. https://www.nice.org.uk/guidance/cg172. 31891465

[5] Sofi F, Macchi C, Abbate R (2014). Mediterranean diet and health status: an updated meta-analysis and a proposal for a literature-based adherence score. Public Health Nutr.

[6] Liese A, Krebs-Smith S, Subar A (2015). The dietary patterns methods project: synthesis of findings across cohorts and relevance to dietary guidance. J Nutr.

[7] Tong TY, Wareham NJ, Khaw KT (2016). Prospective association of the Mediterranean diet with cardiovascular disease incidence and mortality and its population impact in a non-Mediterranean population: the EPIC-Norfolk study. BMC Med.

[8] Behrens G, Fischer B, Kohler S (2013). Healthy lifestyle behaviors and decreased risk of mortality in a large prospective study of U.S. women and men. Eur J Epidemiol.

[9] Prinelli F, Yannakoulia M, Anastasiou CA (2015). Mediterranean diet and other lifestyle factors in relation to 20-year all-cause mortality: a cohort study in an Italian population. Br J Nutr.

[10] Lopez-Garcia E, Rodriguez-Artalejo F, Li TY (2014). The Mediterranean-style dietary pattern and mortality among men and women with cardiovascular disease. Am J Clin Nutr.

[11] Cuenca-Garcia M, Artero EG, Sui X (2014). Dietary indices, cardiovascular risk factors and mortality in middle-aged adults: findings from the aerobics center longitudinal study. Ann Epidemiol.

[12] Knoops KT, de Groot LC, Kromhout D (2004). Mediterranean diet, lifestyle factors, and 10-year mortality in elderly European men and women: the HALE project. JAMA.

[13] Robins J (1986). A new approach to causal inference in mortality studies with a sustained exposure period - application to control of the healthy worker survivor effect. Math Model.

[14] Robins JM, Hernan MA, Brumback B (2000). Marginal structural models and causal inference in epidemiology. Epidemiology.

[15] Milne R L, Fletcher A S, MacInnis R J, Hodge A M, Hopkins A H, Bassett J K, Bruinsma F J, Lynch B M, Dugué P A, Jayasekara H, Brinkman M T, Popowski L V, Baglietto L, Severi G, O’Dea K, Hopper J L, Southey M C, English D R, Giles G G (2017). Cohort Profile: The Melbourne Collaborative Cohort Study (Health 2020). International Journal of Epidemiology.

[16] Ireland P, Jolley D, Giles G (1994). Development of the Melbourne FFQ: a food frequency questionnaire for use in an Australian prospective study involving an ethnically diverse cohort. Asia Pac J Clin Nutr.

[17] Bassett JK, English DR, Fahey MT (2016). Validity and calibration of the FFQ used in the Melbourne collaborative cohort study. Public Health Nutr.

[18] Hodge AM, Simpson JA, Fridman M (2009). Evaluation of an FFQ for assessment of antioxidant intake using plasma biomarkers in an ethnically diverse population. Public Health Nutr.

[19] Hodge AM, Simpson JA, Gibson RA (2007). Plasma phospholipid fatty acid composition as a biomarker of habitual dietary fat intake in an ethnically diverse cohort. Nutr Metab Cardiovasc Dis.

[20] National Health and Medical Research Council of Australia. Australian Guidelines to Reduce Health Risks from Drinking Alcohol. In: Council AGNHaMR: Commonwealth of Australia, Canberra; 2009. https://www.nhmrc.gov.au/about-us/publications/australian-guidelines-reduce-health-risks-drinking-alcohol.

[21] Baghurst K, Record S, Leppard P (2000). Red meat consumption in Australia: intakes, nutrient contribution and changes over time. Aust J Nutr Diet.

[22] MacInnis RJ, Hodge AM, Dixon HG (2013). Predictors of increased body weight and waist circumference for middle-aged adults. Public Health Nutr.

[23] Craig CL, Marshall AL, M. (2003). S. International physical activity questionnaire: 12-country reliability and validity. Med Sci Sports Exerc.

[24] Magliano D, Liew D, Pater H (2003). Accuracy of the Australian National Death Index: comparison with adjudicated fatal outcomes among australian participants in the long-term intervention with pravastatin in Ischaemic disease (LIPID) study. Aust N Z J Public Health.

[25] Lohman TG, Roche AF, Martorell R (1988). Anthropometric standardization reference manual.

[26] Danaei G, Pan A, Hu FB (2013). Hypothetical midlife interventions in women and risk of type 2 diabetes. Epidemiology.

[27] Garcia-Aymerich J, Varraso R, Danaei G (2014). Incidence of adult-onset asthma after hypothetical interventions on body mass index and physical activity: an application of the parametric g-formula. Am J Epidemiol.

[28] Taubman SL, Robins JM, Mittleman MA (2009). Intervening on risk factors for coronary heart disease: an application of the parametric g-formula. Int J Epidemiol.

[29] Dugue PA, Hodge AM, Brinkman MT (2016). Association between selected dietary scores and the risk of urothelial cell carcinoma: a prospective cohort study. Int J Cancer.

[30] Hodge A, Almeida OP, English DR (2013). Patterns of dietary intake and psychological distress in older Australians: benefits not just from a Mediterranean diet. Int Psychogeriatr.

[31] Hodge AM, Bassett JK, Shivappa N (2016). Dietary inflammatory index, Mediterranean diet score, and lung cancer: a prospective study. Cancer Causes Control.

[32] Hodge AM, English DR, Itsiopoulos C (2011). Does a Mediterranean diet reduce the mortality risk associated with diabetes: evidence from the Melbourne collaborative cohort study. Nutr Metab Cardiovasc Dis.

[33] Estruch R, Ros E, Salas-Salvado J (2013). Primary prevention of cardiovascular disease with a Mediterranean diet. N Engl J Med.

[34] Lee AJ, Kane S, Ramsey R, et al. Testing the price and affordability of healthy and current (unhealthy) diets and the potential impacts of policy change in Australia. BMC Public Health. 2016;16. 10.1186/s12889-016-2996-y PMC4828857 27067642

[35] Trichopoulou A, Martinez-Gonzalez MA, Tong TYN, et al. Definitions and potential health benefits of the Mediterranean diet: views from experts around the world. BMC Med. 2014;12. 10.1186/1741-7015-12-112 PMC4222885 25055810

