# Supplementary material for: Sustained adherence to a Mediterranean diet and physical activity on all-cause mortality in the Melbourne Collaborative Cohort Study: application of the g-formula
Source: BMC Public Health. 2019 Dec 26;19:1733. doi: 10.1186/s12889-019-7919-2 (PMC6933918; doi:10.1186/s12889-019-7919-2)
Supplement: Supplementary file 1 — Additional file 1: Figure S1. Causal diagram for study. Figure S2. Flowchart for study participants. Table S1. Summary of time-varying characteristics in study sample. Table S2. Comorbidities and mortality during follow-up. Table S3. Missing data at the three waves. Table S4. Compete case analysis – Cox models for all cause mortality. Table S5. Complete case analysis – All-cause mortality risks using the G-formula. Table S6. G-formula sensitivity analysis (re-ordering time-varying comorbidities). Table S7. G-formula sensitivity analysis (re-ordering time-varying confounders). Table S8. G-formula sensitivity analysis (re-ordering intervention variables). Box S1. Details of imputation approach for missing data. Box S2. Overview of the parametric G-formula. [file 12889_2019_7919_MOESM1_ESM.docx]

Web Figures and Tables

**Contents**

**Web Figure 1**. Causal diagram for study

**Web Figure 2**. Flowchart for study participants

**Web Table 1.** Summary of time-varying characteristics in study sample

**Web Table 2.** Comorbidities and mortality during follow-up

**Web Table 3**. Missing data at the three waves

**Web Table 4**. Compete case analysis – Cox models for all cause mortality

**Web Table 5**. Complete case analysis – All-cause mortality risks using the G-formula

**Web Table 6**. G-formula sensitivity analysis (re-ordering time-varying comorbidities)

**Web Table 7**. G-formula sensitivity analysis (re-ordering time-varying confounders)

**Web Table 8**. G-formula sensitivity analysis (re-ordering intervention variables)

**Web Box 1.** Details of imputation approach for missing data

**Web Box 2.** Overview of the parametric G-formula.

**Web Figure 1**. Causal diagram for study

**
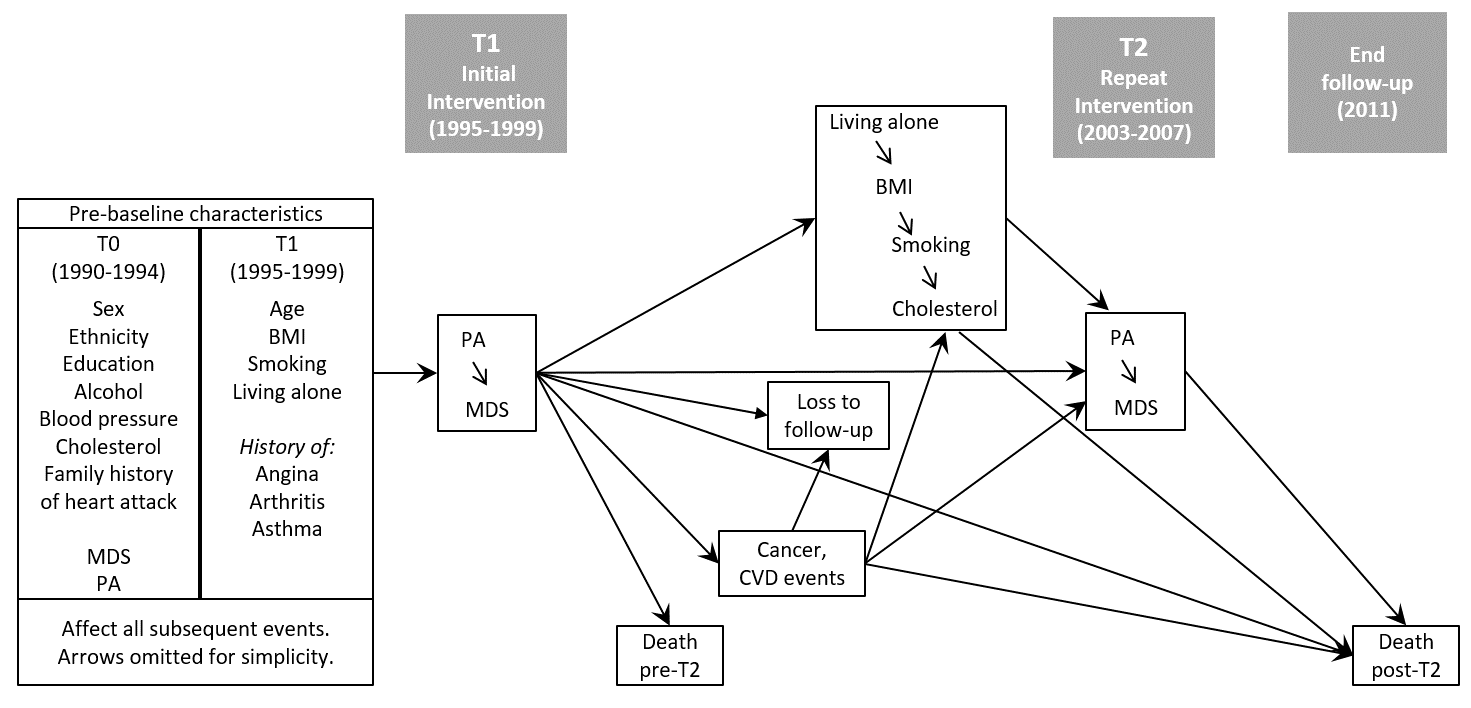
**

**Web Figure 2**. Flowchart for study participants


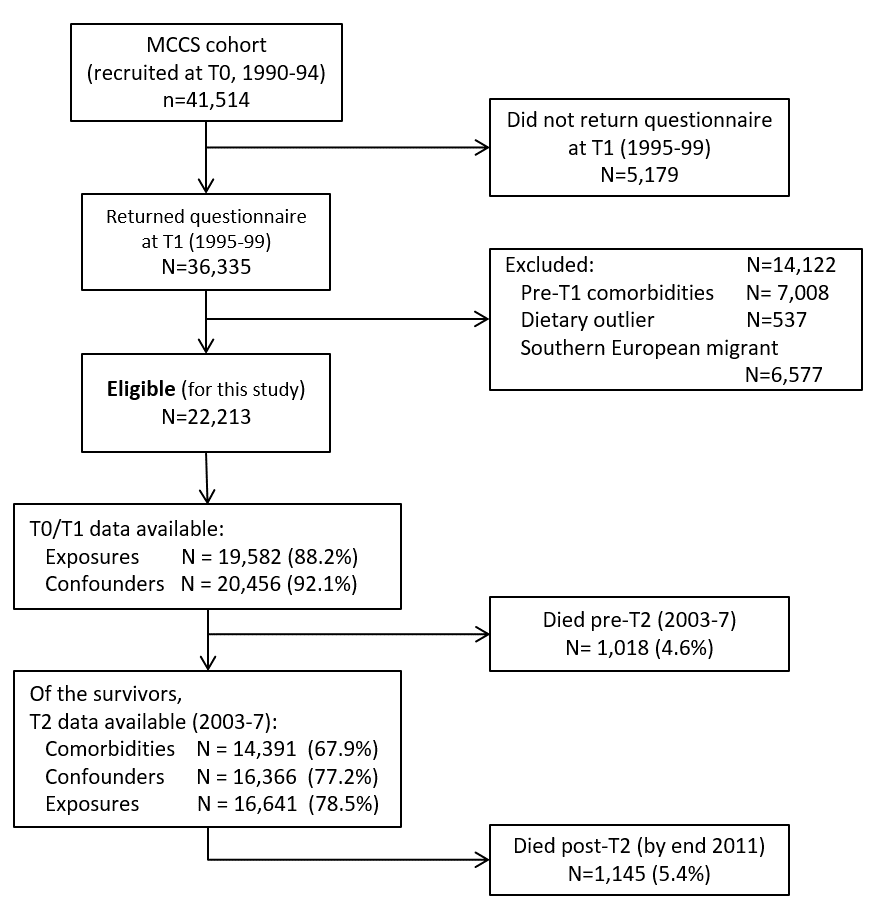


**Web Table 1.** Summary of time-varying characteristics in study sample (n=22,213)

|  | **Women – N (%)** | | | | | | **Men – N (%)** | | | | | |
| --- | --- | --- | --- | --- | --- | --- | --- | --- | --- | --- | --- | --- |
| Timepoint ^a^ | T0 | | T1 | | T2 | | T0 | | T1 | | T2 | |
| Total alive | 13,980 |  | 13,980 |  | 13,469 |  | 8,233 |  | 8,233 |  | 7,726 |  |
| **Exposures** |  |  |  |  |  |  |  |  |  |  |  |  |
| Mediterranean diet score |  |  |  |  |  |  |  |  |  |  |  |  |
| Measured values | 13,980 |  | 12,358 |  | 10,548 |  | 8,233 |  | 7,351 |  | 6,197 |  |
| Low (0-3) | 3,349 | 24.0 % | 4,095 | 33.1 % | 2,893 | 27.4 % | 2,064 | 25.1 % | 3,157 | 42.9 % | 1,941 | 31.3 % |
| Medium (4-5) | 6,148 | 44.0 % | 5,158 | 41.7 % | 4,638 | 44.0 % | 3,660 | 44.5 % | 2,936 | 39.9 % | 2,732 | 44.1 % |
| High (6-9) | 4,483 | 32.1 % | 3,105 | 25.1 % | 3,017 | 28.6 % | 2,509 | 30.5 % | 1,258 | 17.1 % | 1,524 | 24.6 % |
| Physical activity |  |  |  |  |  |  |  |  |  |  |  |  |
| Measured values | 13,980 |  | 13,707 |  | 10,884 |  | 8,233 |  | 8,118 |  | 6,355 |  |
| Low | 4,491 | 32.1 % | 5,073 | 37.0 % | 3,515 | 32.3 % | 2,366 | 28.7 % | 2,661 | 32.8 % | 1,755 | 27.6 % |
| Moderate | 6,033 | 43.2 % | 6,310 | 46.0 % | 4,630 | 42.5 % | 3,177 | 38.6 % | 3,348 | 41.2 % | 2,588 | 40.7 % |
| High | 3,456 | 24.7 % | 2,324 | 17.0 % | 2,739 | 25.2 % | 2,690 | 32.7 % | 2,109 | 26.0 % | 2,012 | 31.7 % |
| **Time-varying confounders** |  |  |  |  |  |  |  |  |  |  |  |  |
| Living arrangements |  |  |  |  |  |  |  |  |  |  |  |  |
| Measured values | 13,980 |  | 13,804 |  | 10,831 |  | 8,233 |  | 8,160 |  | 6,326 |  |
| Living alone | 2,660 | 19.0 % | 2,968 | 21.5 % | 3,205 | 29.6 % | 1,066 | 12.9 % | 1,092 | 13.4 % | 1,033 | 16.3 % |
| Body Mass Index |  |  |  |  |  |  |  |  |  |  |  |  |
| Measured values | 13,974 |  | 12,903 |  | 10,777 |  | 8,230 |  | 7,797 |  | 6,280 |  |
| BMI (kg/m^2^; mean (SD)) | 25.7 | (4.4) | 25.4 | (4.4) | 26.5 | (4.9) | 26.4 | (3.4) | 26.3 | (3.5) | 26.9 | (3.8) |
| Plasma cholesterol |  |  |  |  |  |  |  |  |  |  |  |  |
| Measured values | 13,941 |  |  |  | 10,649 |  | 8,215 |  |  |  | 6,221 |  |
| Cholesterol (mmol/L; mean(SD)) | 5.5 | (1.1) | --- |  | 5.4 | (0.9) | 5.4 | (1.0) | --- |  | 5.0 | (0.9) |
| Smoking status: |  |  |  |  |  |  |  |  |  |  |  |  |
| Measured values | 13,980 |  | 13,966 |  | 11,010 |  | 8,233 |  | 8,228 |  | 6,410 |  |
| Never | 9,155 | 65.5 % | 9,155 | 65.6 % | 7,306 | 66.4 % | 4,063 | 49.4 % | 4,063 | 49.4 % | 3,317 | 51.7 % |
| Former smoker | 3,576 | 25.6 % | 3,622 | 25.9 % | 3,092 | 28.1 % | 3,313 | 40.2 % | 3,349 | 40.7 % | 2,713 | 42.3 % |
| Recent quitter |  |  | 281 | 2.0 % | 228 | 2.1 % |  |  | 207 | 2.5 % | 152 | 2.4 % |
| Current | 1,249 | 8.9 % | 908 | 6.5 % | 384 | 3.5 % | 857 | 10.4 % | 609 | 7.4 % | 228 | 3.6 % |

^a^ T0 = MCCS baseline (considered pre-study baseline for this analysis, 1990-1994), T1 = study baseline for this analysis (1995-1999), T2 = follow-up (2003-2007)

**Web Table 2.** Comorbidities and mortality during follow-up

|  | All | | Women | | Men | |
| --- | --- | --- | --- | --- | --- | --- |
|  | n/N^a^ | % | n/N^a^ | % | n/N^a^ | % |
| **Events between T1 and T2** |  |  |  |  |  |  |
| All-cause mortality | 1,018/22,213 | 4.6 % | 511/13,980 | 3.7 % | 507/8,233 | 6.2 % |
| Self-reported: |  |  |  |  |  |  |
| Heart attack | 352/17,376 | 2.0 % | 145/10,992 | 1.3 % | 207/6,384 | 3.2 % |
| Bypass | 229/17,430 | 1.3 % | 65/11,019 | 0.6 % | 164/6,411 | 2.6 % |
| Angioplasty | 391/17,420 | 2.2 % | 145/11,020 | 1.3 % | 246/6,400 | 3.8 % |
| Stroke | 263/17,394 | 1.5 % | 141/10,997 | 1.3 % | 122/6,397 | 1.9 % |
| Angina | 373/14,511 | 2.6 % | 195/9,212 | 2.1 % | 178/5,299 | 3.4 % |
| Diabetes | 506/14,532 | 3.5 % | 268/9,217 | 2.9 % | 238/5,315 | 4.5 % |
| Cancer diagnosis (registry): |  |  |  |  |  |  |
| All | 1,274/21,195 | 6.0 % | 688/13,469 | 5.1 % | 586/7,726 | 7.6 % |
| Among those with full follow-up data^b^ | 635/11,316 | 5.6 % | 320/7,066 | 4.5 % | 315/4,250 | 7.4 % |
| **Events after T2** |  |  |  |  |  |  |
| All-cause mortality: |  |  |  |  |  |  |
| All | 1,145/21,195 | 5.4 % | 623/13,469 | 4.6 % | 522/7,726 | 6.8 % |
| Among those with full follow-up data^b^ | 471/11,316 | 4.2 % | 241/7,066 | 3.4 % | 230/4,250 | 5.4 % |

^a^ Number with event / Number with that information recorded; ^b^ Full data on PA, MDS, time-varying confounders and comorbidities (i.e. variables used in the G-formula analysis). T0 = initial cohort (MCCS) recruitment (1990-1994), T1 = our study baseline (1995-1999), T2 = follow-up (2003-2007)

**Web Table 3**. Missing data at the three waves.

|  | Number (%) of missing values | | |
| --- | --- | --- | --- |
|  | **Variables measured once** | | |
| **Characteristic &**  **measurement occasion** | Alive at T0/T1  N=22,213 |  | Alive at T2  N=21,195 |
| Sex (T0) | 0 (0) |  |  |
| Age (T1) | 0 (0) | *Post-T1 pre-T2:* |  |
| Highest education (T0) | 0 (0) | Bypass | 4,783 (21.5) |
| Alcohol consumption (T0) | 0 (0) | Angioplasty | 4,793 (21.5) |
| High blood pressure (T0) | 37 (0.1) | Heart attack | 4,837 (21.7) |
| Self-reported Angina (T1) | 21 (0.0) | Stroke | 4,819 (21.6) |
| Self-reported Asthma (T1) | 67 (0.3) | Angina | 7,702 (34.6) |
| Self-reported Arthritis (T0) | 0 (0) | Diabetes | 7,681 (34.5) |
| Family history of heart attack (T0) | 0 (0) | Cancer | 0 (0) |
|  | **Time-updated variables** | | |
|  | Alive at T0  N=22,213 | Alive at T1  N=22,213 | Alive at T2  N=21,195 |
| **Time-varying confounders** |  |  |  |
| Cholesterol | 57 (0.2) | – | 5,343 (25.2) |
| Living alone | 0 (0) | 249 (1.1) | 5,056 (23.8) |
| Body Mass Index | 9 (0.0) | 1,513 (6.8) | 5,156 (24.3) |
| Smoking status | 0 (0) | 19 (0.0) | 4,793 (22.6) |
| **Key exposures** |  |  |  |
| Mediterranean diet score | 0 (0) | 2,504 (11.2) | 5,468 (25.7) |
| Physical activity score | 0 (0) | 388 (1.7) | 4,974 (23.4) |
| **Outcome** |  |  |  |
| All-cause mortality (pre-/post-T2) | – | 0 (0) | 0 (0) |

T0 = initial cohort (MCCS) recruitment (1990-1994), T1 = our study baseline (1995-1999),

T2 = follow-up (2003-2007)

**Web Table 4**. Complete case analysis – Hazard ratios (HR) from Cox models for all cause mortality, for Mediterranean Diet Score (MDS) and Physical Activity (PA), accounting for previous MDS and PA measurements (at T0)

|  | Mediterranean Diet Score | | | | Physical Activity Score | | | |
| --- | --- | --- | --- | --- | --- | --- | --- | --- |
|  | HR | 95% CI | | P | HR | 95% CI | | P |
| Single time-point exposure (T1) |  |  |  |  |  |  |  |  |
| Low | Ref |  |  |  | Ref |  |  |  |
| Medium | 0.90 | (0.81, | 0.99) | 0.032 | 0.87 | (0.79, | 0.96) | 0.005 |
| High | 0.90 | (0.80, | 1.02) | 0.102 | 0.77 | (0.68, | 0.87) | <0.001 |
| + adjusting for previous MDS/PA (T0) |  |  |  |  |  |  |  |  |
| Low | Ref |  |  |  | Ref |  |  |  |
| Medium | 0.92 | (0.83, | 1.02) | 0.115 | 0.89 | (0.80, | 0.98) | 0.016 |
| High | 0.95 | (0.83, | 1.08) | 0.412 | 0.80 | (0.70, | 0.91) | 0.001 |
| Time-updated MDS/PA |  |  |  |  |  |  |  |  |
| Low | Ref |  |  |  | Ref |  |  |  |
| Medium | 0.85 | (0.78, | 0.94) | 0.001 | 0.81 | (0.74, | 0.89) | <0.001 |
| High | 0.84 | (0.75, | 0.94) | 0.002 | 0.73 | (0.65, | 0.82) | <0.001 |
| + adjusting for previous MDS/PA |  |  |  |  |  |  |  |  |
| Low | Ref |  |  |  | Ref |  |  |  |
| Medium | 0.87 | (0.79, | 0.96) | 0.007 | 0.82 | (0.75, | 0.90) | <0.001 |
| High | 0.87 | (0.77, | 0.98) | 0.025 | 0.74 | (0.65, | 0.84) | <0.001 |

T0 = initial cohort (MCCS) recruitment (1990-1994), T1 = our study baseline (1995-1999)

**Web Table 5**. Complete case analysis – Estimated mortality risk under a range of hypothesised interventions on physical activity (PA) and Mediterranean Diet Score (MDS)

|  | | Pre-T2 mortality risk (95% CI)^c^ | | | Post-T2 mortality risk (95% CI)^c^ | | Total^a^ mortality risk (95% CI)^c^ | | Risk difference^b^  (95% CI)^c^ | | Risk Ratio  (95% CI)^c^ | |
| --- | --- | --- | --- | --- | --- | --- | --- | --- | --- | --- | --- | --- |
|  |  | | **Observed risks** | | | | | | | | | |
| Among whole cohort | | 4.43 | | (4.16, 4.69) | 5.08 | (4.79, 5.37) | 9.28 | (8.91, 9.66) |  |  |  |  |
|  |  | | **Estimated risks under no intervention** | | | | | | | | | |
| Natural course | | 4.42 | | (4.15, 4.69) | 4.59 | (4.24, 4.98) | 8.81 | (8.41, 9.28) | Ref |  | Ref |  |
|  |  | | **Estimated risks under hypothetical interventions** | | | | | | | | | |
| **Intervene on everyone** | |  | |  |  |  |  |  |  |  |  |  |
| *Control interventions* | |  | |  |  |  |  |  |  |  |  |  |
| Low PA & low MDS | | 5.56 | | (4.77, 6.31) | 5.35 | (4.05, 6.71) | 10.60 | (9.03, 12.09) | 1.79 | (0.27, 3.05) | 1.20 | (1.03, 1.35) |
| Moderate PA & MDS | | 4.63 | | (4.03, 5.32) | 4.05 | (3.21, 5.03) | 8.50 | (7.45, 9.61) | -0.31 | (-1.27, 0.79) | 0.96 | (0.86, 1.09) |
| *Single interventions* | |  | |  |  |  |  |  |  |  |  |  |
| High PA | | 3.94 | | (3.35, 4.56) | 3.76 | (3.11, 4.45) | 7.55 | (6.73, 8.48) | -1.26 | (-1.98, -0.51) | 0.86 | (0.77, 0.94) |
| High MDS | | 4.47 | | (3.89, 5.08) | 4.21 | (3.48, 4.92) | 8.49 | (7.59, 9.34) | -0.32 | (-1.10, 0.46) | 0.96 | (0.87, 1.05) |
| High PA & MDS | | 4.34 | | (3.30, 5.51) | 3.41 | (2.39, 4.55) | 7.59 | (6.03, 9.09) | -1.22 | (-2.82, 0.32) | 0.86 | (0.68, 1.04) |
| *Repeated interventions* | |  | |  |  |  |  |  |  |  |  |  |
| High PA | | 3.97 | | (3.35, 4.55) | 3.14 | (2.47, 3.80) | 6.99 | (6.15, 7.91) | -1.82 | (-2.61, -1.08) | 0.79 | (0.71, 0.88) |
| High MDS | | 4.48 | | (3.89, 5.06) | 4.05 | (3.22, 4.89) | 8.35 | (7.35, 9.33) | -0.47 | (-1.33, 0.45) | 0.95 | (0.85, 1.05) |
| High PA & MDS | | 4.33 | | (3.27, 5.54) | 3.04 | (1.96, 4.45) | 7.24 | (5.73, 9.05) | -1.57 | (-3.08, 0.24) | 0.82 | (0.65, 1.03) |
| **Intervene on obese only** | |  | |  |  |  |  |  |  |  |  |  |
| *Control interventions* | |  | |  |  |  |  |  |  |  |  |  |
| Low PA & MDS | | 4.58 | | (4.32, 4.92) | 4.75 | (4.33, 5.18) | 9.11 | (8.64, 9.71) | 0.30 | (0.05, 0.58) | 1.03 | (1.01, 1.07) |
| Moderate PA & MDS | | 4.48 | | (4.16, 4.74) | 4.42 | (4.04, 4.82) | 8.70 | (8.25, 9.15) | -0.12 | (-0.34, 0.07) | 0.99 | (0.96, 1.01) |
| *Single interventions* | |  | |  |  |  |  |  |  |  |  |  |
| High PA | | 4.33 | | (4.05, 4.62) | 4.43 | (4.09, 4.84) | 8.57 | (8.14, 9.03) | -0.24 | (-0.39, -0.09) | 0.97 | (0.95, 0.99) |
| High MDS | | 4.41 | | (4.16, 4.73) | 4.58 | (4.18, 4.93) | 8.78 | (8.38, 9.25) | -0.03 | (-0.21, 0.10) | 1.00 | (0.98, 1.01) |
| High PA & MDS | | 4.40 | | (4.05, 4.72) | 4.39 | (4.02, 4.80) | 8.60 | (8.11, 9.14) | -0.21 | (-0.50, 0.05) | 0.98 | (0.94, 1.01) |
| *Repeated interventions* | |  | |  |  |  |  |  |  |  |  |  |
| High PA | | 4.35 | | (4.06, 4.61) | 4.26 | (3.91, 4.61) | 8.43 | (7.97, 8.84) | -0.39 | (-0.62, -0.26) | 0.96 | (0.93, 0.97) |
| High MDS | | 4.42 | | (4.17, 4.72) | 4.51 | (4.10, 4.91) | 8.73 | (8.31, 9.23) | -0.08 | (-0.29, 0.12) | 0.99 | (0.97, 1.01) |
| High PA & MDS | | 4.40 | | (4.04, 4.72) | 4.21 | (3.87, 4.69) | 8.42 | (8.01, 8.99) | -0.39 | (-0.67, -0.06) | 0.96 | (0.92, 0.99) |

T0 = initial cohort (MCCS) recruitment (1990-1994), T1 = our study baseline (1995-1999), T2 = follow-up (2003-2007)

^a^ Estimated as: Pr(Dead) = Pr(Dead pre-T2) + Pr(Dead post-T2|Alive at T2) x Pr(Alive at T2); ^b^ Compared with natural course assuming no loss to follow-up.

^c^ 95% percentile bootstrap confidence intervals using 499 bootstrap samples; ^d^ Death post-T2 estimated using participants followed-up at T2. This is the estimator we would obtain if outcome data were available only on followed-up participants, as is often the case.

**Web Table 6**. Sensitivity analysis re-ordering time-varying comorbidities (point estimates only) – Estimated mortality risk under a range of hypothesised interventions on physical activity (PA) and Mediterranean Diet Score (MDS)

|  | Pre-  T2 | Post-  T2 | Total^a^ | Risk  diff^b^ | Risk  Ratio | Pre-  T2 | Post-  T2 | Total^a^ | Risk  diff^b^ | Risk  Ratio |
| --- | --- | --- | --- | --- | --- | --- | --- | --- | --- | --- |
|  | **Observed risks** | | | | | **Estimated risks under no intervention** | | | | |
| Natural course | 4.58 | 5.40 | 9.74 |  |  | 4.58 | 5.54 | 9.86 | Ref | Ref |
|  | **Intervene on everyone** | | | | | **Intervene on obese only** | | | | |
| *Control interventions* |  |  |  |  |  |  |  |  |  |  |
| Repeated low PA and low MDS | 5.67 | 7.67 | 12.91 | 3.04 | 1.31 | 4.71 | 5.84 | 10.27 | 0.41 | 1.04 |
| Repeated moderate PA and MDS | 4.53 | 4.65 | 8.97 | -0.89 | 0.91 | 4.56 | 5.32 | 9.64 | -0.23 | 0.98 |
| Repeated low PA | 5.14 | 7.10 | 11.88 | 2.01 | 1.20 | 4.66 | 5.72 | 10.11 | 0.25 | 1.03 |
| Repeated low MDS | 4.62 | 6.37 | 10.69 | 0.83 | 1.08 | 4.60 | 5.66 | 10.00 | 0.13 | 1.01 |
| *Single interventions* |  |  |  |  |  |  |  |  |  |  |
| Initial high PA | 4.32 | 4.91 | 9.02 | -0.85 | 0.91 | 4.53 | 5.42 | 9.71 | -0.15 | 0.98 |
| Initial MDS | 4.71 | 5.14 | 9.61 | -0.26 | 0.97 | 4.58 | 5.49 | 9.82 | -0.05 | 1.00 |
| Initial PA and MDS | 4.65 | 4.32 | 8.77 | -1.10 | 0.89 | 4.56 | 5.34 | 9.65 | -0.21 | 0.98 |
| *Repeated interventions* |  |  |  |  |  |  |  |  |  |  |
| Repeated high PA | 4.33 | 4.36 | 8.50 | -1.36 | 0.86 | 4.54 | 5.29 | 9.59 | -0.28 | 0.97 |
| Repeated high MDS | 4.71 | 4.69 | 9.19 | -0.68 | 0.93 | 4.59 | 5.36 | 9.71 | -0.15 | 0.98 |
| Repeated high PA and MDS | 4.64 | 3.52 | 8.00 | -1.87 | 0.81 | 4.57 | 5.13 | 9.47 | -0.40 | 0.96 |

**Web Table 7**. Sensitivity analysis re-ordering time-varying confounders (point estimates only) – Estimated mortality risk under a range of hypothesised interventions on physical activity (PA) and Mediterranean Diet Score (MDS)

|  | Pre-  T2 | Post-  T2 | Total^a^ | Risk  diff^b^ | Risk  Ratio | Pre-  T2 | Post-  T2 | Total^a^ | Risk  diff^b^ | Risk  Ratio |
| --- | --- | --- | --- | --- | --- | --- | --- | --- | --- | --- |
|  | **Observed risks** | | | | | **Estimated risks under no intervention** | | | | |
| Natural course | 4.58 | 5.40 | 9.74 |  |  | 4.57 | 5.51 | 9.83 | Ref | Ref |
|  | **Intervene on everyone** | | | | | **Intervene on obese only** | | | | |
| *Control interventions* |  |  |  |  |  |  |  |  |  |  |
| Repeated low PA and low MDS | 5.67 | 7.68 | 12.92 | 3.08 | 1.31 | 4.70 | 5.85 | 10.28 | 0.44 | 1.04 |
| Repeated moderate PA and MDS | 4.52 | 4.67 | 8.98 | -0.85 | 0.91 | 4.57 | 5.32 | 9.64 | -0.19 | 0.98 |
| Repeated low PA | 5.16 | 7.08 | 11.88 | 2.05 | 1.21 | 4.64 | 5.73 | 10.10 | 0.27 | 1.03 |
| Repeated low MDS | 4.63 | 6.37 | 10.70 | 0.87 | 1.09 | 4.59 | 5.65 | 9.98 | 0.15 | 1.01 |
| *Single interventions* |  |  |  |  |  |  |  |  |  |  |
| Initial high PA | 4.32 | 4.91 | 9.02 | -0.82 | 0.92 | 4.53 | 5.43 | 9.72 | -0.12 | 0.99 |
| Initial MDS | 4.71 | 5.14 | 9.60 | -0.23 | 0.98 | 4.59 | 5.48 | 9.82 | -0.01 | 1.00 |
| Initial PA and MDS | 4.65 | 4.32 | 8.77 | -1.07 | 0.89 | 4.58 | 5.33 | 9.67 | -0.17 | 0.98 |
| *Repeated interventions* |  |  |  |  |  |  |  |  |  |  |
| Repeated high PA | 4.32 | 4.36 | 8.49 | -1.34 | 0.86 | 4.52 | 5.28 | 9.56 | -0.27 | 0.97 |
| Repeated high MDS | 4.71 | 4.70 | 9.19 | -0.64 | 0.93 | 4.59 | 5.38 | 9.72 | -0.11 | 0.99 |
| Repeated high PA and MDS | 4.65 | 3.51 | 7.99 | -1.85 | 0.81 | 4.56 | 5.13 | 9.46 | -0.38 | 0.96 |

**Web Table 8**. Sensitivity analysis re-ordering intervention variables (point estimates only) – Estimated mortality risk under a range of hypothesised interventions on physical activity (PA) and Mediterranean Diet Score (MDS)

|  | Pre-  T2 | Post-  T2 | Total^a^ | Risk  diff^b^ | Risk  Ratio | Pre-  T2 | Post-  T2 | Total^a^ | Risk  diff^b^ | Risk  Ratio |
| --- | --- | --- | --- | --- | --- | --- | --- | --- | --- | --- |
|  | **Observed risks** | | | | | **Estimated risks under no intervention** | | | | |
| Natural course | 4.58 | 5.40 | 9.74 |  |  | 4.58 | 5.54 | 9.86 | Ref | Ref |
|  | **Intervene on everyone** | | | | | **Intervene on obese only** | | | | |
| *Control interventions* |  |  |  |  |  |  |  |  |  |  |
| Repeated low PA and low MDS | 5.65 | 7.67 | 12.89 | 3.03 | 1.31 | 4.72 | 5.85 | 10.29 | 0.43 | 1.04 |
| Repeated moderate PA and MDS | 4.54 | 4.66 | 8.98 | -0.88 | 0.91 | 4.57 | 5.31 | 9.64 | -0.22 | 0.98 |
| Repeated low PA | 5.10 | 7.03 | 11.77 | 1.91 | 1.19 | 4.63 | 5.74 | 10.10 | 0.24 | 1.02 |
| Repeated low MDS | 4.65 | 6.47 | 10.82 | 0.95 | 1.10 | 4.59 | 5.67 | 10.00 | 0.13 | 1.01 |
| *Single interventions* |  |  |  |  |  |  |  |  |  |  |
| Initial high PA | 4.33 | 4.93 | 9.04 | -0.82 | 0.92 | 4.52 | 5.44 | 9.72 | -0.14 | 0.99 |
| Initial MDS | 4.70 | 5.05 | 9.51 | -0.35 | 0.96 | 4.59 | 5.47 | 9.80 | -0.06 | 0.99 |
| Initial PA and MDS | 4.65 | 4.33 | 8.78 | -1.08 | 0.89 | 4.58 | 5.34 | 9.68 | -0.19 | 0.98 |
| *Repeated interventions* |  |  |  |  |  |  |  |  |  |  |
| Repeated high PA | 4.34 | 4.39 | 8.54 | -1.32 | 0.87 | 4.52 | 5.30 | 9.58 | -0.28 | 0.97 |
| Repeated high MDS | 4.70 | 4.59 | 9.08 | -0.78 | 0.92 | 4.58 | 5.34 | 9.68 | -0.18 | 0.98 |
| Repeated high PA and MDS | 4.64 | 3.51 | 7.99 | -1.87 | 0.81 | 4.56 | 5.24 | 9.56 | -0.31 | 0.97 |

**Web Box 1.** Details of imputation approach used for missing data

| Missing data | | |
| --- | --- | --- |
| **1. Missing data description**  Web Table 3 describes the missing data at the three study waves.  **2. Differences between those with complete and incomplete data**  Web Table 2 shows that participants lost to follow-up are likely to be sicker than those followed-up. However, after taking their fully observed all-cause mortality status into account, the assumption of missing at random (i.e. that given all observed information, missing values themselves are not related to the probability of that value being missing) is more likely to be satisfied.  **3. Multiple imputation details**  We applied multiple imputation, assuming data were missing at random. A fully-conditional specification (chained equations) approach was applied, using the imputation models listed below. We created 5 imputed datasets. Computational considerations prevented a larger number of imputed datasets from being used. The analysis (e.g. G-formula) was applied to each imputed dataset and then Rubin’s rules were used to combine estimates. T2 values were imputed conditional on survival to T2. | | |
| **Fully conditional specification imputation models** | | |
| *Imputed variable* | *Imputation model* | *Derived variables*  *(passive imputation)* |
| Physical activity score (T1, T2)  Mediterranean diet score (T1,T2) | Predictive mean matching  (5 nearest neighbours) | Categorised physical activity and Mediterranean diet scores (low, medium high) |
| BMI (T0,T1,T2), Cholesterol (T0, T2) | Linear regression | Grouped BMI and cholesterol (5 categories) |
| High blood pressure (T0),  Asthma (T1), Angina (T1) | Logistic regression | – |
| Living alone (T1, T2) | Logistic regression | Combined with previous living status (recently alone, long term alone, etc.). |
| Post T1 and pre-T2: bypass, angioplasty, stroke, heart attack, diabetes, and angina. | Logistic regression | – |
| Smoking status (T1) | Ordinal logistic regression | – |
| Quitting post-T1 pre-T2 | Logistic regression | Smoking status (T2) |
| Fully observed variables included as predictors in all imputation models^a^:  Sex, age (T1), educational status, living alone (T0), arthritis (T0), family history of heart attack, alcohol consumption (T0), Mediterranean diet score (T0), physical activity score (T0), cancer diagnosis pre-T1 and post-T2, all-cause mortality (pre-T2 and post-T2). | | |
| ^a^ Except: reduced set of predictors included in imputation model for quitting smoking at T2 due to convergence issues | | |

| **Web Box 2.** Overview of the parametric G-formula.  T0 is the pre-study baseline, T1 is the study baseline, T2 is the follow-up visit. | |
| --- | --- |
| **Step 1: Parametric regression models** | **Step 2: Monte Carlo simulation** |
| **1a. Fit models for the outcome pre- and post-T2**  **Pre-T2 all-cause mortality** was modelled using logistic regression conditional on physical activity and Mediterranean diet scores at T1 and their interaction, the scores at T0, and all measured confounders at T0 and T1 (listed above).  **Post-T2 all-cause mortality** was modelled similarly, with the model restricted to survivors at T2 who had full follow-up data, and using all prior diet and activity scores and their interactions, all measured confounders at T0 and T1, measured time-varying confounders (listed in 1c), and comorbidities (1b). The most recent value of BMI, with the change since the last measurement, was used rather than include both highly-correlated measurements. Smoking status was included, distinguishing between recent quitters and long-term quitters using the previous measure of smoking status.  **1b. Fit models for each pre-T2 comorbidity**  **Cancer, heart attacks, angioplasty, bypass, stroke, diabetes and angina** were each modelled using logistic regression on physical activity and Mediterranean diet scores and their interaction (at T1), previous values of the scores, and all measured confounders at T0 and T1. The model for cancer included all participants, models for all other comorbidities were restricted to those with full follow-up T2 data since information on these was obtained at T2. Comorbidities were modelled in the order listed, with all comorbidities included in subsequent models as explanatory variables.  **1c. Fit models for each time-varying confounder**  Time-varying confounders – **living alone, BMI, smoking status and total plasma cholesterol** –were modelled, in the listed order. Living alone and quitting smoking (assuming no participant begins smoking at this age) were modelled using logistic regression on all prior physical activity and diet scores, an interaction between the most recent diet and activity scores, all measured confounders at T0 and T1, intervening comorbidities (1b) and prior time-varying confounder values (e.g. the model for BMI at T2 included living alone at T2, but not cholesterol or smoking at T2). BMI and cholesterol were modelled using linear regression.  **1d. Fit models for intervention**  *[Required for natural course only]*  **Mediterranean diet and physical activity** scores were each modelled as three-level ordinal variables using ordinal logistic regression. Models included all prior values of diet and activity scores with an interaction between the most recent diet and activity score, all measured confounders at T0 and T1, comorbidities (1b) and time-varying confounder values (1c). The model for physical activity at T2 included the diet score at T2, but not vice versa (under the assumed causal ordering of diet then physical activity).  **1e. Fit models for censoring (loss to follow-up)**  *[Required for natural course only]*  **Loss to follow-up** was modelled using logistic regression including all prior diet and activity scores, their interaction, all measured confounders at T0 and T1, and pre-T2 cancer diagnosis. | All parts of step 2 are undertaken separately for each hypothetical intervention considered.  **2a. Intervene at T1**  The observed T1 confounder information was retained for all participants, modifying the T1 intervention values (physical activity and Mediterranean diet scores) in accordance with the specified intervention (e.g. for the intervention “set T1 physical activity to the highest level” we would replace all participants’ T1 activity scores by ‘high’).  **2b. Simulate pre-T2 mortality**  We used the estimated regression coefficients from (1a) to predict the probability of pre-T2 all-cause mortality. We compared these to a value randomly drawn from a standard uniform distribution and set the participant’s outcome to dead if the random value was below the predicted probability, and alive otherwise.  **Step 2c. Simulate comorbidities and confounders at T2**  For participants simulated to remain alive at T2, we used the estimated regression coefficients from (1b) and (1c) to simulate values for the intervening comorbidities (in the order listed in 1b) and time-varying confounders: living alone, BMI, total plasma cholesterol and smoking status. For continuous variables (BMI, plasma), the simulated value was the predicted value from the regression coefficients plus a random error term drawn from a normal distribution with mean zero and variance equal to the mean-squared error from the fitted model. For the remaining variables, values were simulated by comparing the predicted probability of a particular confounder value to a value randomly drawn from a standard uniform distribution.  **Step 2d. Simulate post-T2 mortality**  As for step 2b, restricted to participants who were simulated to remain alive at T2.  **Step 2e. Estimate cumulative mortality risk**  Cumulative mortality risk was estimated as: $p_{m}=p_{m(T1)}+\left( 1-p_{m\left( T1 \right)} \right)p_{m(T2)}$, where $p_{m(T1)}$ is the estimated mortality from T1 to T2, estimated as the proportion of participants simulated as being dead at T2, and $p_{m(T2)}$is the estimated mortality post T2 among survivors at T2, estimated as the proportion of simulated survivors at T2 simulated to die post-T2.  All steps thus far were repeated 50 times. The average cumulative mortality was taken across the 50 runs to minimise Monte Carlo error.  **2f. Bootstrap**  Complete case: All previous steps were repeated in 500 non-parametric bootstrap samples. 95% bootstrap percentile confidence intervals for the cumulative mortality were obtained.  Imputed data: All previous steps were repeated in 100 non-parametric bootstrap samples of participants within each of 5 imputed datasets. 95% bootstrap normal-based confidence intervals for the cumulative mortality were obtained.  *[Computational time precluded larger numbers of imputed datasets or bootstrap samples.]* |
|  | **Step 3: Exposure effects** |
|  | **3a. Risk Ratios and Risk Differences**  Risk ratios and risk differences were obtained by comparing estimated risks with the risk estimated under the natural course.  **3b. Population Attributable Fractions**  PAF was calculated as: (I_0_-I_1_)/I_0_, where I_0_ is the risk under the natural course and I_1_ is the risk under the hypothesised intervention.  For all estimates, bootstrap confidence intervals were obtained as described in step 2f. |
